# COVID-19 pandemic in India: challenges and silver linings

**DOI:** 10.1136/postgradmedj-2020-137780

**Published:** 2020-04-16

**Authors:** Sagarika Kamath, Rajesh Kamath, Prajwal Salins

**Affiliations:** School of Management, Manipal University, Manipal, Karnataka, India; Prasanna School of Public Health, Manipal University, Manipal, Karnataka, India; Department of Health Information Management, Manipal College of Health Professions, Manipal Academy of Higher Education, Manipal, Karnataka, India

**Keywords:** epidemiology, health economics

India faces multiple major challenges on the COVID-19 front. It is densely populated: 464 people/km^2^ compared with Italy’s 206, Spain’s 91, Iran’s 52 and the USA’s 36. It has a huge population: 1380 million (USA 330 million, Iran 83 million, Italy 60 million, Spain 46 million). Social distancing without total shutdowns is unthinkable, especially in the big cities with crowded streets, trains, buses and offices. Cough hygiene is largely absent. Hand hygiene is equally suspect. The latest data from the government National sample survey organisation say that only 36% of Indians wash their hands with soap before a meal.^1^ Even more distressingly, 160 million Indians do not have access to clean water to wash their hands.^2^ The research suggests that diabetes and hypertension worsen COVID-19 outcomes: the prevalence among Indian adults of diabetes and hypertension is 10% and 25%, respectively. India has high rates of TB and pneumonia. People have resisted being screened and flouted quarantines with impunity.^3^ The awareness about disease dynamics is very poor, even among the wealthier and more educated parts of the population: after the national voluntary ‘people’s curfew’ called by the prime minister on 22 March, which was by and large a success, people came out on the streets and celebrated with no attention to social distancing, achieving the exact opposite of what the curfew was supposed to achieve. Imposing a lockdown is next to impossible in India’s vast rural hinterland, home to 900 million people (65% of the population). Seventy per cent of the rural population is dependent on agriculture and April and May is the harvesting season for their Rabi crops.

Before the 21-day lockdown imposed from 25 March, mathematical modelling put the number of expected cases in India between 300 million and 500 million by July end, with a peak somewhere in April and/or May of 100 million cases, with 10 million (10%) requiring hospitalisation, 5 million recovering, 5 million needing critical care and 1 to 2.5 million deaths. After the lockdown, these numbers are estimated to come down by about 80%, with 1 million needing critical care, including ventilator support.^4^ India has just 20 000 ventilators. *That is a 98% shortfall*. The government response has been to make 40 000 more by June. Even into late March, India had an abnormally low testing rate of 18 per million population (South Korea 6931, Italy 5268, UK 1469, USA 1280).^5^ While there are several reasons for this, it is clear that this strategy is unsustainable and that testing rates will have to be raised dramatically. Media reports indicate that the acute shortage of personal protective equipment (PPE) is of the government’s own making. While industry bodies had reached out to the government with the need to create stockpiles of PPE in early February, the government did not ban exports of PPE until mid-March. Bureaucratic delays compounded matters further.^6^

The COVID-19–induced 21-day lockdown has put more strain on an economy that was already experiencing declining growth and increased joblessness. More than 75% of India’s substantial 100 million migrant workers have lost their jobs overnight. The retail industry is expected to lose 10 million jobs, the restaurant industry 1.5 million jobs and the transport industry 5 million jobs. Fifteen million unregistered workers have been left out of a meagre government benefits package. There is no doubt that with falling incomes, this economic pandemic will increase the proportion of health expenditure that is catastrophic, thereby pushing more people below the poverty line. The COVID-19 pandemic will also result in a decrease in accessibility to healthcare as many private care providers, both individuals and institutions pull themselves out of service, both in public interest and as a measure for self preservation: this is already being seen with the postponement of elective surgeries and the closure of OPDs in several geographies. Although temporary, it is sure to cause significant morbidity and mortality from non–COVID-19 causes. This will result in an increase in out-of-pocket expenditure on healthcare and will probably worsen non–COVID-19 health outcomes as well, given the twin predicaments of a COVID-19–induced depletion of healthcare manpower and the COVID-19 burden on the healthcare system. India’s chronically underfunded public health system and an unaccountable private healthcare system will make things even more difficult. These are fertile areas for future research.

India’s first case was confirmed on 30 January 2020. Italy and Spain had their first cases confirmed a day later. The USA had its first case confirmed on 20 January. After almost 2 months, India has had only 20 COVID-19 confirmed deaths as against 9000 in Italy, 5000 in Spain and 1200 in the USA. There is a big question mark on the accuracy of the Indian COVID-19 mortality data in the absence of comprehensive testing of severe acute respiratory infections (SARIs). India sees 100 million cases of influenza-like illness every year, which have the same symptoms as COVID-19. That works out to an average of 27 000 cases per day. Into the final week of March, India was testing about 2000 cases per day (much higher than the 100 tests a day in mid-March): *a deficit of 92%*. With a mortality rate of 0.1%, the 100 million influenza cases cause 100 000 deaths every year: 273 every day. In the absence of any data on how many of these fatalities were tested for COVID-19, the possibility of underdetection of COVID-19 deaths exists. Going on, surveillance for cases of SARI would need to be strengthened to build confidence in the COVID-19 mortality data.

India has just 0.8 doctors per 1000 population as against Italy’s 4.1, China’s 1.8, Spain’s 4.1, Iran’s 1.1 and the USA’s 2.6.^7^ India has just 0.7 hospital beds per 1000 population as against Italy’s 3.4, Spain’s 3, Iran’s 1.5 and the USA’s 2.9. In the wake of a tsunami of COVID-19 cases requiring hospitalisation, this manpower and infrastructure are going to prove inadequate. There is some hope in India that the avalanche will not come. This hope stems from two silver linings: the weather and the age demographic of the population. There is some evidence that the COVID-19 virus is weather and humidity sensitive. The spread of outbreaks of COVID-19 suggest a preference for cooler and drier climates.^8^ India’s summer temperatures average 32–40°C with a humidity range of 50%–70% across the country. If COVID-19 actually is weather and humidity sensitive, it would slow down the spread of the virus in India. Only 6% of India’s population is above 65 years of age compared with Italy’s 22%, Spain’s 18% and the USA’s 16%. With case fatality rates for COVID-19 significantly lower among younger populations, this augurs well for India. As the COVID-19 story of death and economic destruction unspools over the next few months, Indians will be hoping that the two silver linings deliver some respite.
